# Supplementary material for: Formation of bridgmanite-enriched layer at the top lower-mantle during magma ocean solidification
Source: Nat Commun. 2020 Jan 28;11:548. doi: 10.1038/s41467-019-14071-8 (PMC6987212; doi:10.1038/s41467-019-14071-8)
Supplement: Supplementary file 1 — Supplementary Information [file 41467_2019_14071_MOESM1_ESM.docx]

Supplementary Information for “Formation of bridgmanite-enriched layer at the top lower-mantle during Magma Ocean solidification” by Xie et al.

Supplementary Note 1: Densification mechanisms in the different pressure ranges

Supplementary Table 1: Summary of experimental conditions and results

Supplementary Table 2: Mean composition on quenched Fo sample of MA23

Supplementary Table 3: Model for the melt viscosity using dimensionless temperature (Eq. (3))

Supplementary Table 4: End-member contents of various mantle compositions (Molar ratio)

Supplementary Table 5: Physical parameters for a deep magma ocean on Earth

Supplementary Figure 1: Experimental set-up used for the measurement of melt viscosity at high pressures

Supplementary Figure 2: Viscosities of silicate melts under pressure

Supplementary Figure 3: Error analysis of Run S3219 by Monte Carlo simulation

Supplementary Figure 4: Correlation between viscosity profiles and densification mechanisms

Supplementary Figure 5: Activation enthalpy of the silicate melts viscosity

Supplementary Figure 6: Comparison of viscosity of Fa and Fo liquid at 2500 K

Supplementary Figure 7: Major controlling parameters for the MO

Supplementary Figure 8: Solid and liquid densities in the MO

Supplementary Figure 9: Solid and liquid densities at the bottom of MO

Supplementary Figure 10: Crystal/critical diameter ratio (ignoring grain growth due to Ostwald ripening)

Supplementary Figure 10: Crystal/critical diameter ratio (using Fe partition coefficient equals 0.2 or using ferropericlase as solid phase at lower mantle depths)

**Supplementary Note 1**

**Densification mechanisms in the different pressure ranges.** The relatively complex pressure dependence measured in this study (Supplementary Fig. 4) can be related to the densification mechanisms. Based on molecular dynamic simulation^22^, three distinct densification mechanisms (T1, T2 and T3) were proposed before the coordination number of Si changes upon compression of sodium silicate melts. At the lowest pressures (T1 mechanism), silicate melts behave like ionic liquids consisting of glass-structure modifier cations (Mg or Ca) and SiO_4_ groups; The main densification mechanism is the change in coordination number (CN) of modifier cations and this is expected to induce an increase of the melt viscosity with increasing the pressure. Within the T2 region, the main densification mechanism is the collapse of the SiO_4_ network; this compression mechanism a negative pressure dependence of the viscosity, due to the bending of Si–O–Si structural units or possibly the increasing concentration of five-fold Si-O coordination species, as suggested by NMR spectroscopy on quenched glasses^24^ and molecular dynamics calculations^23^. This compression mechanism is also accompanied by a continuous, progressive, change of the CN of modifier cations. Then, in the T3 region, the silicate melt structure gradually evolves toward a coesite-like network through an increasing number of four-membered tetrahedral rings and a decreasing number of five to seven-membered rings; It yields an increase of the melt viscosity with increasing pressure, while the cation CN remains almost constant.

Altogether, the pressure dependence of the viscosity of Fo, En and Di melts can be interpreted based on the progressive evolution between these three densifications mechanisms (Supplementary Fig. 4a, b, and c). They are T2 (0 to ~10 GPa) and T3 (10 to 30 GPa) for Fo-melt; T2 (0 to ~10 GPa) and T3 (~10 to ~30 GPa) for En-melt and T1 (0 to ~5 GPa), T2(~5 to ~21 GPa) and T3(~21 to ~30 GPa) for Di-melt. Comparison between En and Di melts suggests that higher pressures are required to induce the change of densification mechanisms from T1 to T2, and T2 to T3, when the modifier-cation is larger (Supplementary Fig. 4d).

**Supplementary Table 1. ׀ Summary of experimental conditions and results.**

| **Run #** | **Sample** | **P (GPa)** | **T (K)** | **𝑑s (µm)** | **𝒗_s_ (mm s^-1^)** | ***ƞ* (mPa s)** |
| --- | --- | --- | --- | --- | --- | --- |
| **MA23** | Fo | 5.7±0.7 | 2420±100 | 76±2 | 1.641±0.006 | 27±1.4 |
| **MA19** | Fo | 9.6±0.7 | 2573±100 | 130±2 | 4.3±0.02 | 21±0.4 |
| **MA30** | Fo | 12.7±0.7 | 2700±100 | 60±2 | 1.05±0.02 | 25.8±1.6 |
| **MA28** | Fo | 20.07±0.04 | 2793±100 | 59±2 | 0.823±0.006 | 32±2 |
| **MA24** | Fo | 23.9±0.6 | 2873±100 | 67±2 | 0.747±0.003 | 44±3 |
| **MA26** | Fo | 29±1 | 3000±150 | 60±2 | 0.566±0.003 | 48±3 |
| **S3259** | En | 4.83±0.3 | 2203±50 | 67±2 | 0.901±0.004 | 40.2±2.1 |
| **MA35** | En | 5.7±1 | 2450±100 | 70±2 | 1.44±0.01 | 26.9±1.4 |
| **S3170** | En | 8.8±1 | 2453±150 | 144±2 | 5.05±0.03 | 20.4±0.4 |
| **S3171** | En | 8.8±0.4 | 2473±50 | 83±2 | 2.2±0.02 | 21.6±1.7 |
| **S3172** | En | 14.7±0.5 | 2645±50 | 76±2 | 3.1±0.1 | 12.8±0.8 |
| **S3175** | En | 15.3±1 | 2623±100 | 68±2 | 2.58±0.05 | 12.9±0.7 |
| **S3219** | En | 24.1±0.5 | 2836±90 | 70±2 | 1.85±0.01 | 19.1±0.9 |
| **S3220** | En | 29.9±0.7 | 3250±100 | 67±2 | 2.69±0.02 | 12±0.6 |
| **S3256** | Di | 5±1 | 2453±100 | 73±2 | 1.67±0.01 | 23.9±1.1 |
| **S3257** | Di | 4.76±0.3 | 2303±50 | 72±2 | 0.915±0.006 | 42.8±2.1 |
| **S3260** | Di | 5±1 | 2313±50 | 70±2 | 0.91±0.006 | 40.4±2 |
| **MA31** | Di | 5.7±1 | 2400±100 | 67±2 | 1.57±0.01 | 23.1±1 |
| **S3173** | Di | 9.9±0.7 | 2400±150 | 143±2 | 1.75±0.04 | 58±2 |
| **MA34** | Di | 12.6±0.7 | 2500±100 | 73±2 | 1.198±0.005 | 31.9±2 |
| **MA32** | Di | 20.25±1 | 2550±100 | 65±2 | 1.89±0.007 | 16.3±1 |
| **S3267** | Di | 23±1 | 2903±100 | 67±2 | 3.44±0.02 | 9.4±0.5 |
| **S3269** | Di | 23±1 | 2793±100 | 77±2 | 3.21±0.04 | 12.7±0.5 |
| **S3291** | Di | 26±0.8 | 2903±100 | 69±2 | 3.5±0.1 | 9.7±0.5 |

Run numbers starting from “S” or “MA” specify experiments performed at beamline BL04B1 (SPring-8, Japan) or Psiché, (SOLEIL, France), respectively. “𝑑s” is the diameter of a sphere at ambient pressure.

**Supplementary Table 2. ׀ Mean composition on quenched Fo sample of MA23.** Data is given in oxide wt% along with the standard deviation (δ).

| **Oxide** | **wt%** | **δ** |
| --- | --- | --- |
| **MgO** | 58.3 | 0.3 |
| **Al_2_O_3_** | 0.003 | 0.003 |
| **WO_3_** | 0.04 | 0.05 |
| **ReO_2_** | 0.05 | 0.045 |
| **SiO_2_** | 42.7 | 0.2 |
| **Total** | 101.093 |  |

**Supplementary Table 3. ׀ Model for the melt viscosity using dimensionless temperature (Eq. (3)).**

| **Parameters of broken line fits ^a^** | | | | | | |
| --- | --- | --- | --- | --- | --- | --- |
| **Composition** | ***η_0_* (Pa s)** | ***a_0_*** | ***a_1_* (GPa^-1^)** | ***b_0_*** | ***b_1_* (GPa^-1^)** | ***Po* (GPa)** |
| **Mg_2_SiO_4_** | 2.3(12) x 10^-4^ | 5.12 (56) | -0.062 (1) | 4.14((52) | 0.043(5) | 9.65 |
| **MgSiO_3_** | 7.63(484) x 10^-5^ | 8.45 (70) | -0.33 (6) | 5.23 (70) | 0 | 9.65 |
| **CaMgSi_2_O_6_** | 3.17(685) x 10^-8^ | 17 (2) | -0.263 (8) | 12.8(20) | 0 | 18 |
| **Fe_2_SiO_4_^+^** | 7.5(37) x 10^-3^ | 2.22(53) | -0.0965(177) | 0 | 0 |  |
| **Parameters of third order polynomial fits ^b^** | | | | | | |
| **Composition** | ***η_0_* (Pa s)** | ***c_0_*** | ***c_1_* (GPa^-1^)** | ***c_2_* (GPa^-2^)** | ***c_3_* (GPa^-3^)** | |
| **Mg_2_SiO_4_** | 1.72 (160) x 10^-4^ | 5.43 (104) | -0.128 (24) | 9.17 (179) x 10^-3^ | -1.55 (37) x 10^-4^ | |
| **MgSiO_3_** | 1.49 (45) x 10^-5^ | 10.35 (34) | -0.645 (23) | 3.74 (27) x 10^-2^ | -6.7 (7) x 10^-4^ | |
| **CaMgSi_2_O_6_** | 8.9 (15) x 10^-8^ | 16.4 (18) | -0.346(55) | 7.2 (54) x 10^-3^ | -2.1(14) x 10^-5^ | |

**a.** Linear fits are conducted for the two sections independently, as $E_{a}^{*}\left( P \right)=\left\{ \begin{aligned} a_{0}+a_{1}P, P\leq P_{0} \\ {b_{0}+b}_{1}P, P>P_{0} \end{aligned} \right.$.

**b.** Third order polynomial fits: $E_{a}^{*}\left( P \right)=c_{0}+c_{1}P+c_{2}P^{2}+c_{3}P^{3}$.

+. Parameters of Fe_2_SiO_4_ are fitted based on experimental data up to 10 GPa from ref. 9.

**Supplementary Table 4. ׀ End-member contents of various mantle compositions (Molar ratio).**

| **Endmembers** | **Fo** | **En** | **Di** | **An** |
| --- | --- | --- | --- | --- |
| **Chondrite^33^** | 31.77 | 61.9 | 1.95 | 4.4 |
| **KLB-1^57^** | 63.26 | 32.7 | 0.7 | 3.32 |

**Supplementary Table 5. ׀ Physical parameters for a deep magma ocean on Earth**

| **Heat capacity, c_p_** | 1803 (KLB-1) J kg^-1^ K^-1^  1725 (Chondrite) J kg^-1^ K^-1^ |
| --- | --- |
| **Gravity, g** | 10 m s^-2^ |
| **Earth radius, R_Earth_** | 6.37×10^6^ m |
| **Apparent surface energy, σ_app_** | 0.02 J m^-2^ **^ref.^** ^5^ |
| **Angular velocity, Ω** | 10^-4^ s^-1^ **^ref.^** ^5^ |


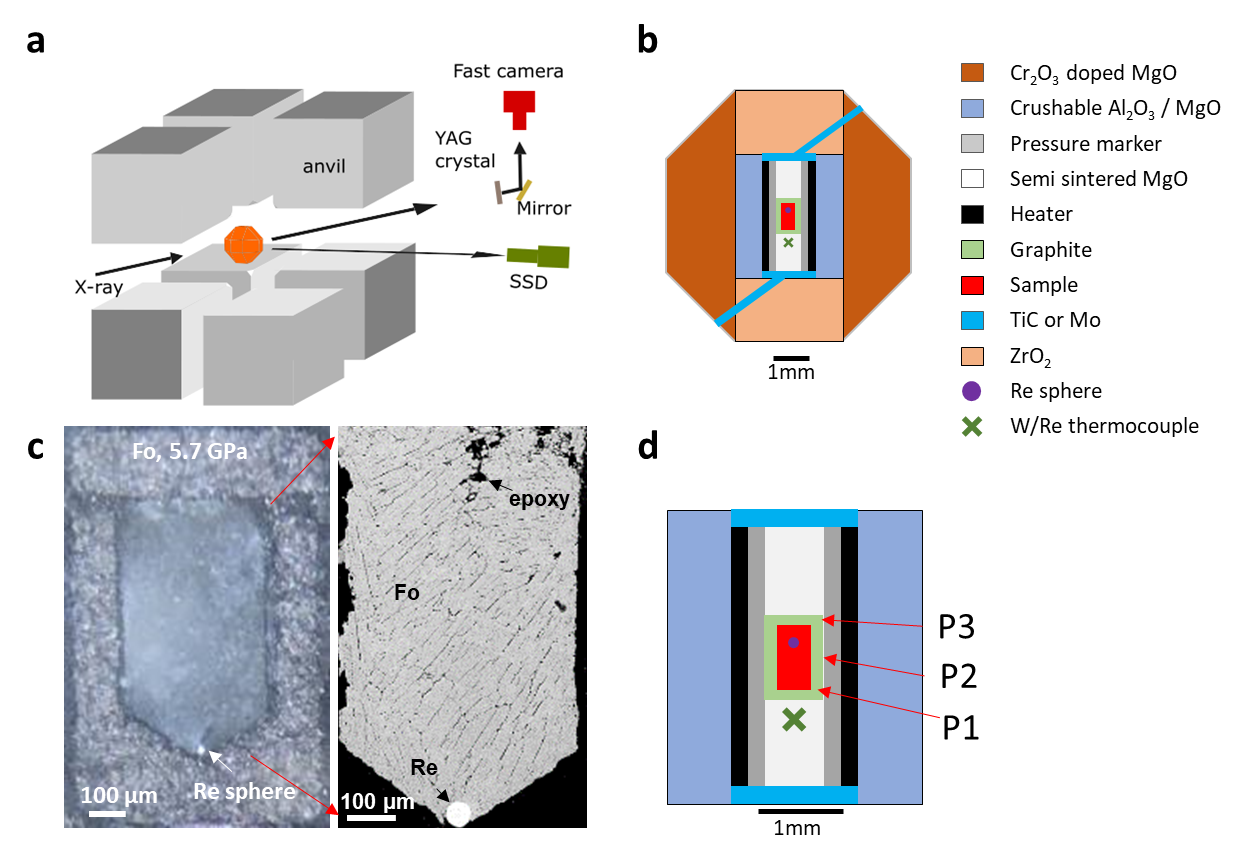


**Supplementary figure 1: Experimental set-up used for the measurement of melt viscosity at high pressures: a,** At synchrotron facilities, the germanium solid-state detector (SSD) or the fast camera are used to performed X-ray diffraction or X-ray radiography, respectively. **b,** Schematic cross-sections of octahedron. **c,** Optical (left) and back scanning electron (right) images of recovered samples in run MA23. Re sphere was observed at the bottom of the sample after its fall through the melt at high pressure and temperature. No sign of reaction between Re sphere and melt was observed. Size of the recrystallized Fo grains is homogeneous in the whole capsule. **d,** Schematic sketch illustrating our method to determine the temperature gradient using X-ray diffraction; Volume of the MgO-pressure marker recorded at several positions (P1-P3) in the cell assembly evidences a temperature difference of less than 60 K from the thermocouple to the sample center (see text).

**
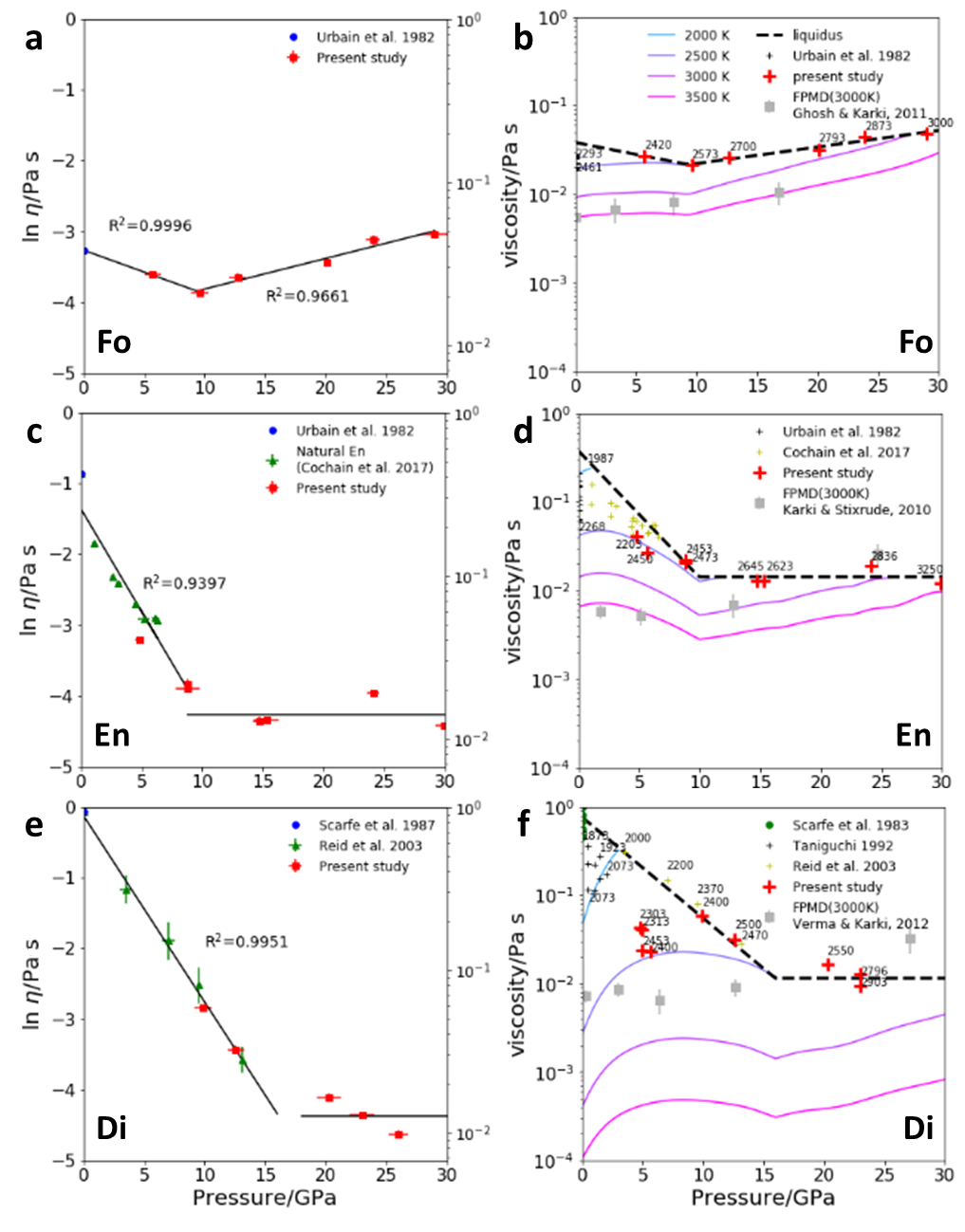
**

**Supplementary figure 2: Viscosities of silicate melts under pressure**. Upper (**a**, **b**), middle (**c**, **d**) and lower (**e**, **f**) frames correspond to Fo, En and Di compositions, respectively. Left columns **(a, c, e)**: Viscosities of silicate melts along the liquidus. Symbols indicate experimental data. The experimental uncertainties are within the symbol sizes. Linear fits were conducted along two distinct sections (Supplementary Table 3). Right columns **(b, d, f)**: Solid lines indicate viscosities estimated along isotherms based on Eq. (1). Dashed line indicates viscosities along liquidus. Numbers annotated near symbols refer to corresponding temperatures. FPMD: first-principle molecular dynamic simulation results.


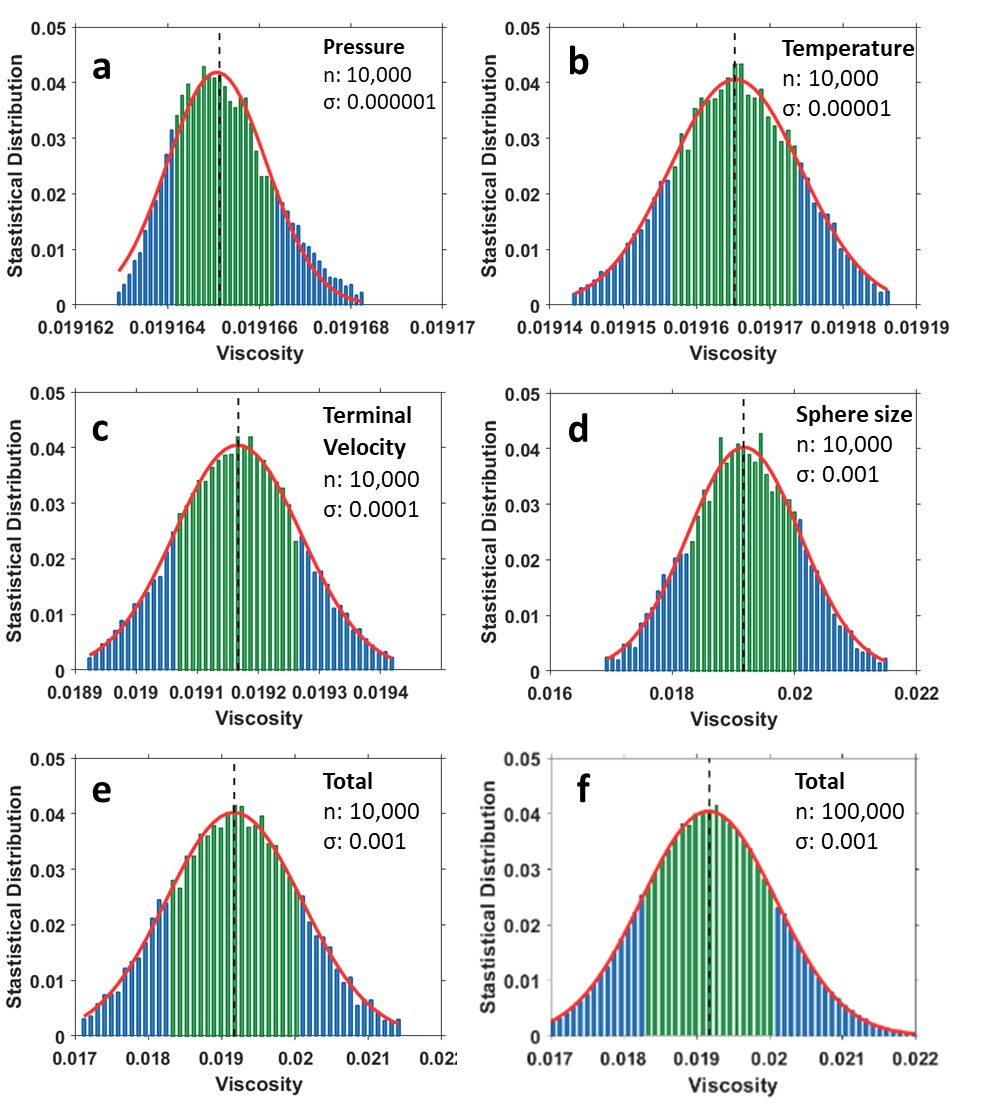


**Supplementary figure 3: Error analysis of Run S3219 by Monte Carlo simulation. a**, **b**, **c,** **d,** uncertainty on viscosity obtained from the propagation of uncertainties from pressure, temperature, terminal velocity and sphere size, respectively. The 1σ standard deviations of four parameters used for simulation are reported in Table 1. **e**, **f**, total error distributions with sampling number of 10,000 and 100,000, respectively.

**
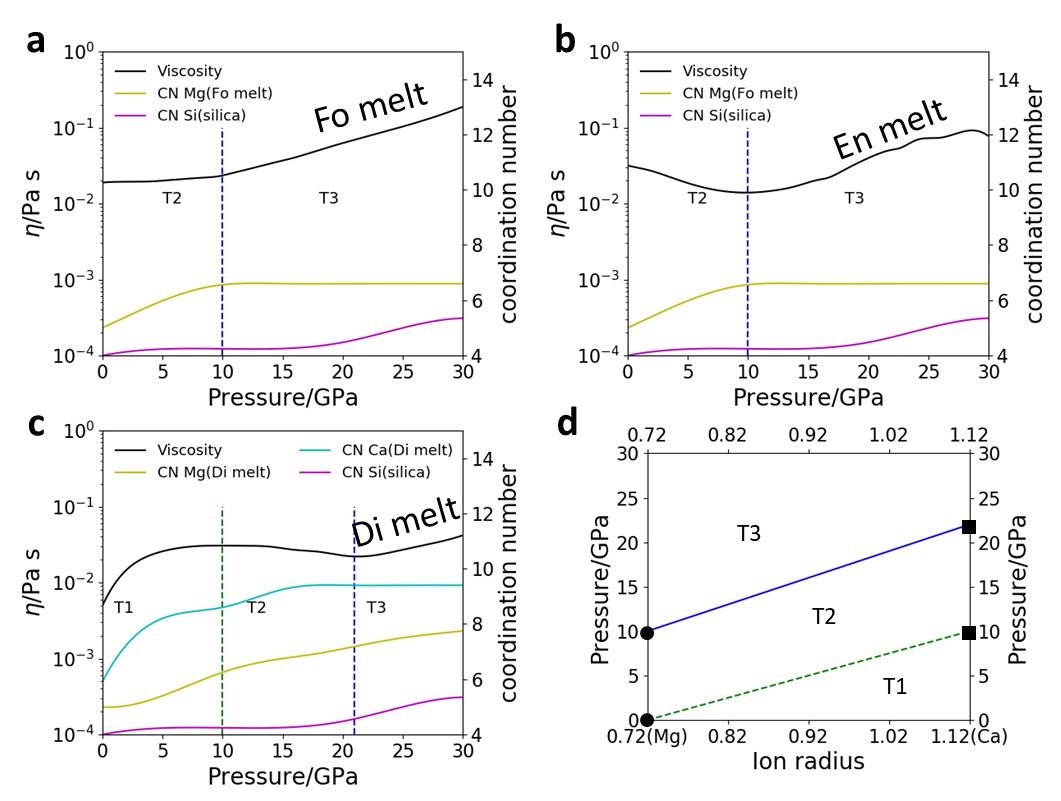
**

**Supplementary figure 4: Correlation between viscosity profiles and densification mechanisms.** Viscosity profiles for (**a**) Fo, (**b**) En and (**c**) Di melt at 2500 K (black curve) are compared with pressure domains where different densification mechanisms (T1, T2 and T3) have been previously reported^22^. The red, yellow and blue curves report the evolution with pressure of the coordination number of Si, Mg and Ca, respectively^S1 and references therein^. (**d**) Change of densification mechanism as a function of pressure and ionic radius along the Mg to Ca substitution. The T1 to T2 transition pressure is assumed to be 0 for the En-melt.

**
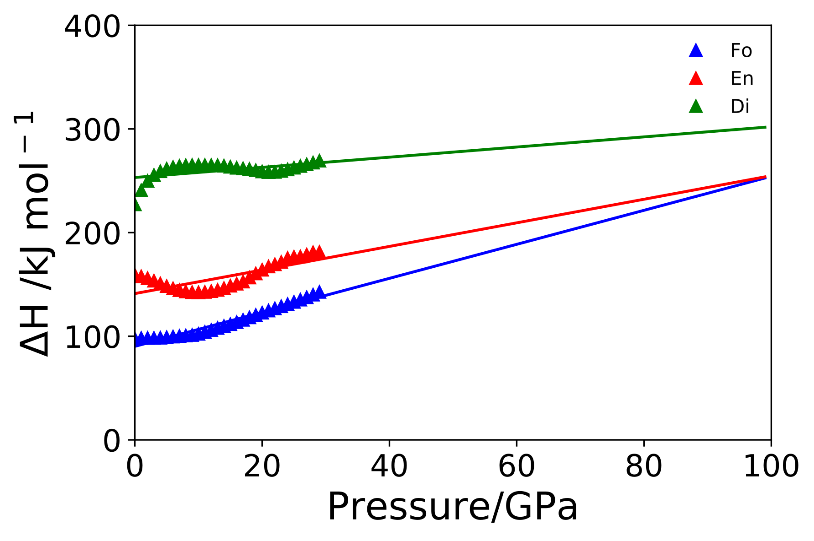
**

**Supplementary figure 5: Activation enthalpy of the silicate melts viscosity.** Blue, red and green lines indicate the evolution with pressure of the activation enthalpy of melts viscosity of Fo, En and Di compositions, respectively. The complex evolutions observed for En and Di at low pressures are fitted by straight lines to enable the extrapolation to mantle pressures higher than 30 GPa based on the Arrhenius law.

**Supplementary figure 6: Comparison of viscosity of Fa and Fo liquid at 2500 K.** Viscosities of both Fo and Fa melt were calculated using Eq. (1) with parameters in Supplementary Table 2.


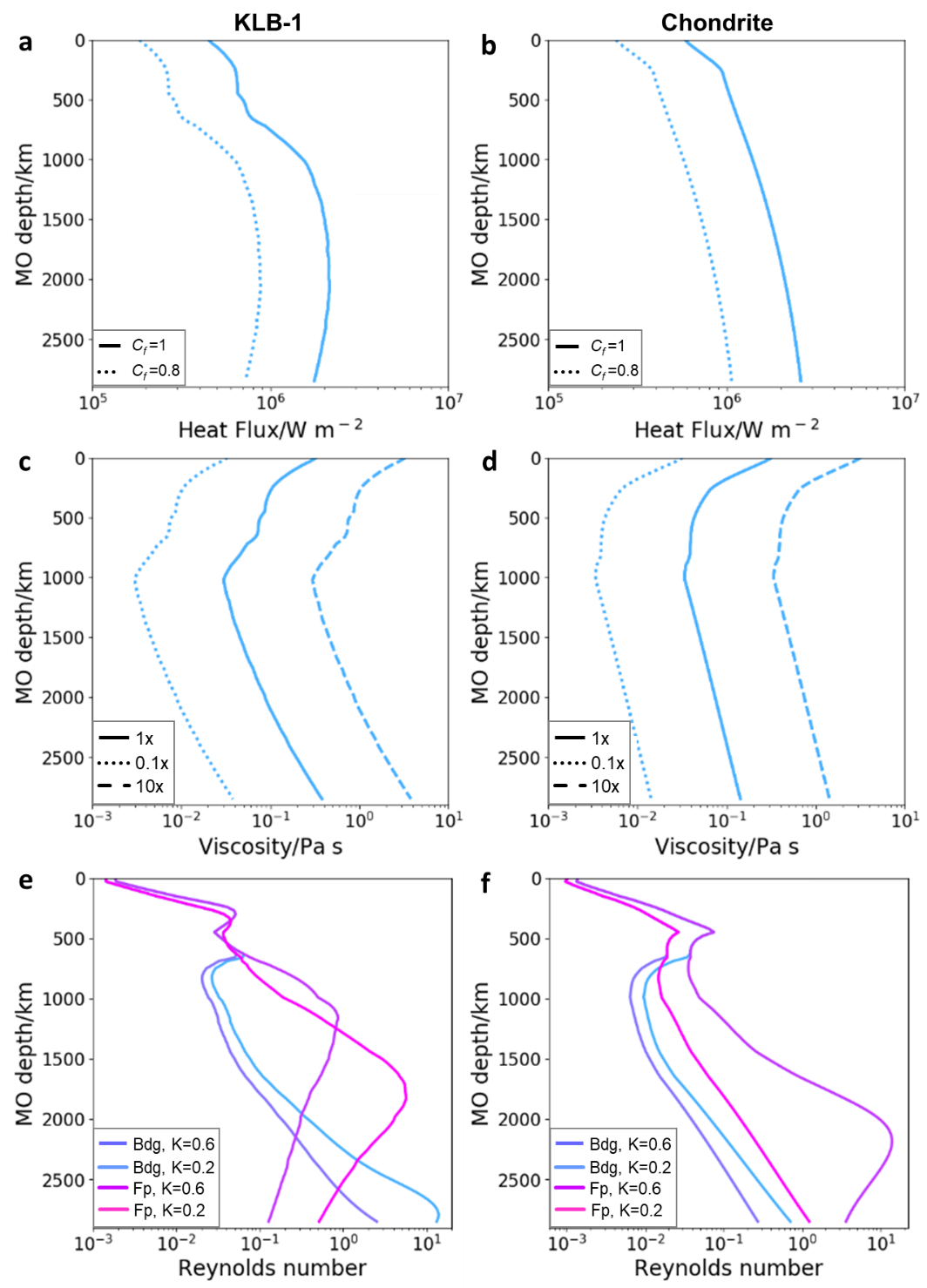


**Supplementary Figure 7: Major controlling parameters for the MO. Left (a, c, e) and right (b, d, f) columns** correspond to peridotitic and chondritic-type compositions, respectively. **Upper frames (a, b):** Heat flux though the MO. Solid and dotted lines correspond to thermal radiation at the Earth's surface for a surface temperature equal to the mantle potential temperature (*C_f_* =1, Eq. (16)) and for an effective temperature 20 % lower (*C_f_* =0.8), respectively. **Middle frames (c, d)**: averaged viscosity along adiabatic curve in a 1000 km thick crystallization zone. A local minimum appears at ~1000 km, which favors fractional solidification. **Lower frames (e, f)**: Reynolds number for grains of the critical size suspended in the MO. K: Fe partition coefficient between solid and melt; Bdg and Fp refer to a solid phase composed of bridgmanite and ferropericlase, respectively.


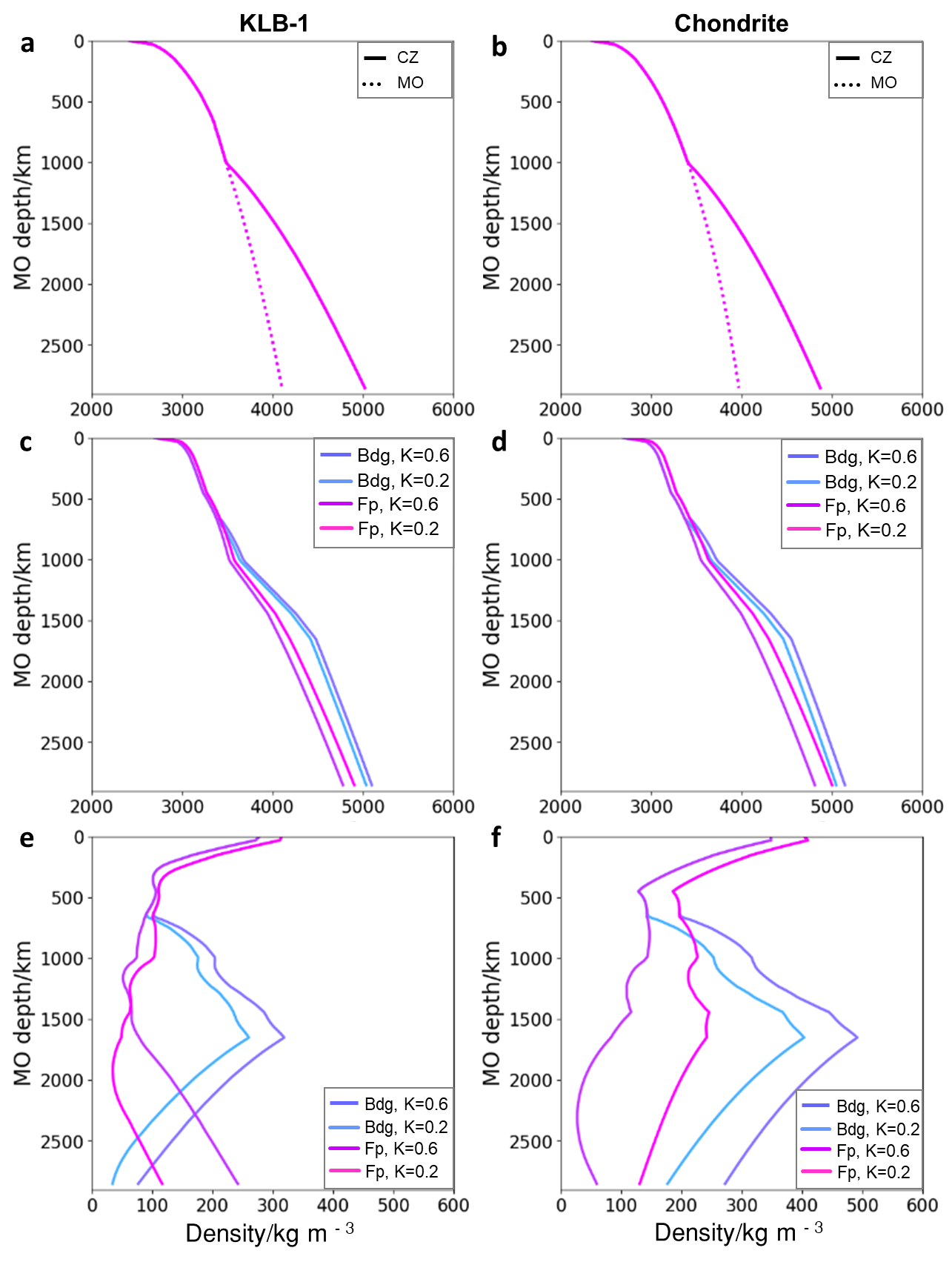


**Supplementary Figure 8: Solid and liquid densities in the MO:** **Left (a, c, e) and right (b, d, f) columns** for peridotitic and chondritic-type compositions, respectively. **Upper frames (a, b):** Solid or dotted lines correspond to melt density along adiabatic curve averaged over a crystallization zone extending 1000 km above the MO-bottom (CZ), or over the entire MO (MO), respectively. **Middle frames (c, d):** Density of solid along adiabatic curve averaged in CZ. **Lower frames (e, f):** Density contrast along adiabatic curve averaged in CZ. The local maximum found at ~1500 km favors fractional solidification. K: partition coefficient between solid and melt, Bdg and Fp: refer to a solid phase composed of bridgmanite and ferropericlase, respectively.


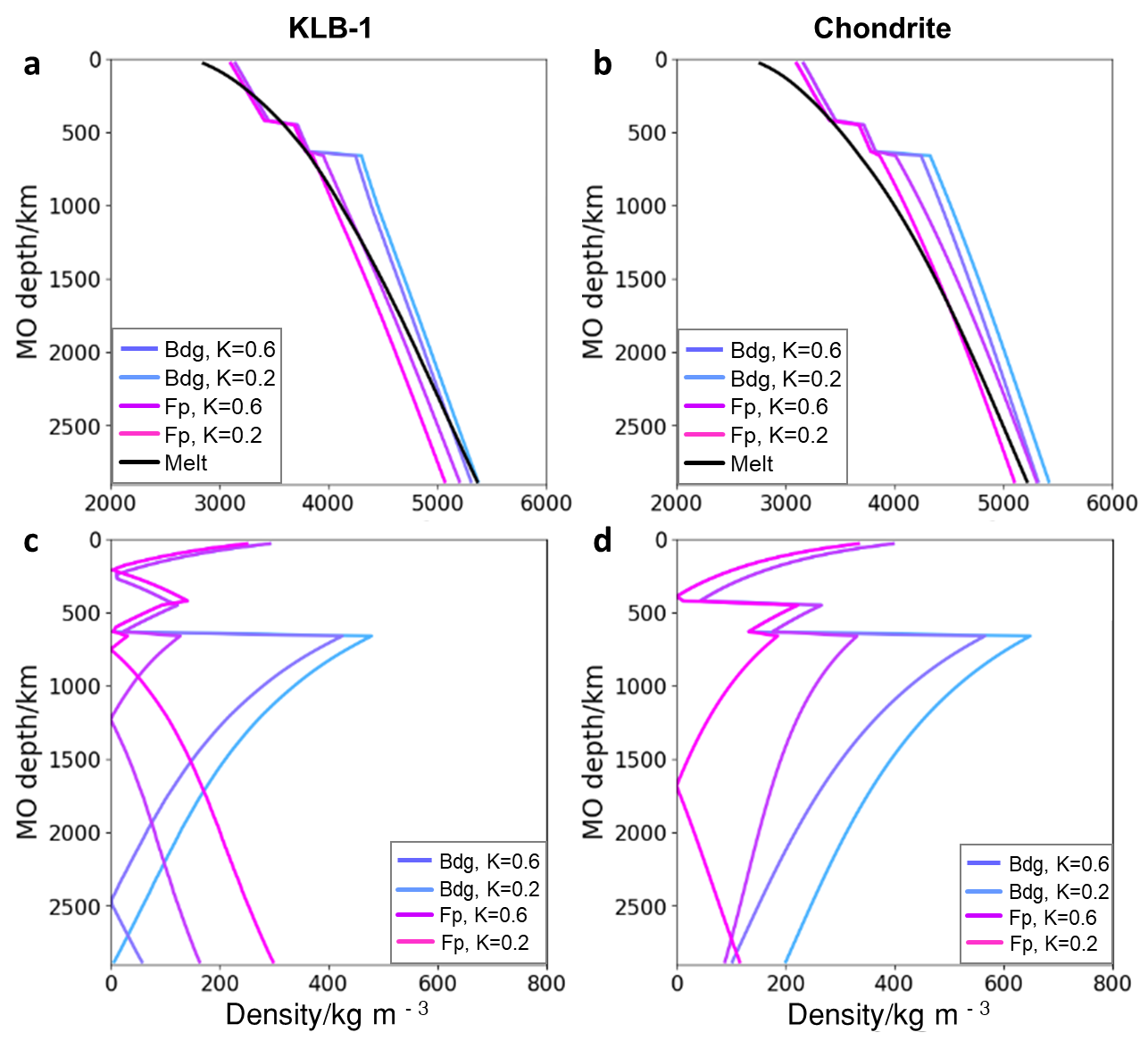


**Supplementary Figure 9: Solid and liquid densities at the bottom of MO:** **Left (a, c) and right (b, d) columns** for peridotitic and chondritic-type compositions, respectively. **Upper frames (a, b):** Density of solid and melt at the bottom of MO. **Lower frames (c, d):** Density contrast at the bottom of MO. The local maximum found at 660 and 450 km due to the first liquidus phase change from bridgmanite to majorite and from majorite to olivine, respectively. K: partition coefficient between solid and melt, Bdg and Fp: refer to a solid phase composed of bridgmanite and ferropericlase, respectively.

**Supplementary Figure 10: Crystal/critical diameter ratio**: Same as Fig. 4 but ignoring grain growth due to Ostwald ripening. **Left column (a, c, e):** KLB-1 composition. **Right column (b, d, f):** chondritic-type mantle composition.

**Supplementary Figure 11: Crystal/critical diameter ratio**: same as Fig. 4 but using Fe partition coefficient equals 0.2 **(a, b)** or using ferropericlase as solid phase at lower mantle depths **(c, d).** **Left column (a, c):** KLB-1 composition. **Right column (b, d):** chondritic-type mantle composition.
