# Supplementary material for: Formation of bridgmanite-enriched layer at the top lower-mantle during magma ocean solidification
Source: Nat Commun. 2020 Jan 28;11:548. doi: 10.1038/s41467-019-14071-8 (PMC6987212; doi:10.1038/s41467-019-14071-8)
Supplement: Supplementary file 3 — Description of Additional Supplementary Files [file 41467_2019_14071_MOESM3_ESM.pdf]

Description of Additional Supplementary Files

File name: Supplementary Movie 1
Description: An example video of sphere falling process (S3267). a, Images of the sphere recorded by contrast of X-ray absorption at a speed of 1000 frames per second. b, Integrated signal of X-ray absorption. A maximum denotes the sphere position. c, Sphere position as a function of time during an entire fall duration of less than 200 ms. The sphere falls with a constant velocity over more than 50% of the total distance of ~500 μm, which allows the determination of the melt viscosity.
